# Bone marrow sarcoidosis in Takayasu arteritis: Case report and literature review

**DOI:** 10.1097/MD.0000000000051482

**Published:** 2026-11-06

**Authors:** Oussama Fakih, Sara Hachem, Racha Chamas, Sally Abi Dargham, Ouidade Aitisha Tabesh, Fouad Fayad

**Affiliations:** a Department of Rheumatology, Lebanese Hospital Geitaoui-UMC, Faculty of Medical Sciences – Lebanese University, Beirut, Lebanon; b Department of Internal Medicine, Division of Rheumatology, Lebanese American University Gilbert and Rose-Marie Chagoury School of Medicine, Byblos, Lebanon.

**Keywords:** bone marrow, case report, granulomatous disorder, sarcoidosis, Takayasu arteritis

## Abstract

**Rationale:**

Takayasu arteritis (TA) is a systemic granulomatous large-vessel vasculitis predominantly involving the aorta and its primary branches. Sarcoidosis is a systemic granulomatous disorder characterized histologically by non-necrotizing epithelioid granulomas, most commonly affecting the lungs. Although the coexistence of TA and sarcoidosis has been documented, bone marrow involvement by sarcoidosis in a patient with concomitant TA has not, to our knowledge, been previously reported.

**Patient concerns:**

A 46-year-old woman with a confirmed diagnosis of TA presented with elevated systemic inflammatory markers and diffuse hypermetabolic activity on positron emission tomography (PET). Clinical, radiological, and pathological evaluations were performed.

**Diagnoses:**

Bone marrow biopsy revealed multiple non-necrotizing granulomas, supporting bone marrow involvement by sarcoidosis. This overlap of granulomatous conditions was identified in the context of persistent systemic inflammation.

**Interventions:**

Mycofenolate Mofetil was replaced by Infliximab therapy.

**Outcomes:**

The patient exhibited significant clinical improvement and marked improvement in inflammatory markers with infliximab therapy.

**Lessons:**

This case highlights the importance of maintaining a broad differential diagnosis in patients with autoimmune or inflammatory diseases who develop new systemic manifestations. Recognizing rare organ involvement in overlapping granulomatous conditions is critical for accurate diagnosis and management.

## 1. Introduction

Takayasu arteritis (TA) is a systemic granulomatous large-vessel vasculitis that predominantly affects women of childbearing age. The disease occurs worldwide, with the highest prevalence reported among Asian populations.^[1]^ The aorta and its major branches, especially the coronary, subclavian, carotid, and renal arteries, are the vessels primarily involved. Pathophysiology involves immune cell infiltration, causing arterial wall thickening, stenosis, and aneurysm formation.^[2]^ This macrophage-driven inflammation initially targets the vessel’s adventitia and media, progressing to panarteritis. Cardiac and vascular manifestations include diminished or absent pulses, giving the condition its nickname “pulseless disease,” claudication, hypertension, and blood pressure discrepancies, particularly between the upper limbs.^[3]^

Sarcoidosis is a systemic granulomatous disease characterized by non-necrotizing epithelioid granulomas. It can affect virtually any organ, although the lungs, skin, eyes, and lymph nodes are the most commonly involved,^[4]^ resulting in widely variable clinical presentations. Bone marrow involvement is rare. The incidence and prevalence of sarcoidosis show significant geographical and ethnic variation, reflecting differences in genetic susceptibility across age, sex, and race. The pathogenesis of sarcoidosis is complex; it involves an interplay between genetic predisposition and environmental triggers that results in a dysregulated immune response predominantly mediated by CD4+ T lymphocytes.^[5]^ Activated CD4+ T cells accumulate and release inflammatory cytokines, contributing to granuloma formation.

Multiple cases of coexisting Takayasu arteritis and sarcoidosis have been reported in the literature, suggesting an association between the 2 diseases. However, bone marrow involvement in such cases has not been previously documented. This article presents a case of concurrent Takayasu arteritis and sarcoidosis with bone marrow involvement in a 46-year-old Middle Eastern woman, discussing the clinical presentation, potential association, and implications for disease management and outcomes.

## 2. Case report

A 46-year-old woman, diagnosed with TA in 2007, was being treated with tocilizumab 800 mg (Actemra, Genentech, San Francisco) monthly and mycophenolate mofetil 2 g daily. She presented to the clinic for further evaluation of abnormal biopsy findings from an esophagogastroduodenoscopy and ileocolonoscopy performed to investigate recent-onset microcytic anemia and fatigue. Gastric biopsies revealed chronic gastritis with multiple granulomas. Biopsies from the ileum also revealed submucosal lesions, each measuring up to 4 mm.

Upon presentation, the patient’s vital signs were normal. Her TA history included myocardial infarction, treated with coronary artery bypass graft (CABG) and metallic aortic valve replacement. The patient reported no new neurological, respiratory, abdominal, or urinary symptoms. Physical examination showed multiple punctate, erythematous, blanching, non-pruritic lesions on the upper extremities, cervical region, and upper thorax. Lung and abdominal auscultation were unremarkable. A systolic murmur was audible over the aortic area. Joint range of motion was within normal limits.

Laboratory testing revealed microcytic anemia with decreased ferritin levels, elevated inflammatory markers, leukopenia, and abnormal liver function tests, as summarized in Table 1. Other laboratory parameters were within normal limits. Magnetic resonance angiography (MRA) of the neck, thorax, and abdomen demonstrated total occlusion of the left subclavian artery and right vertebral artery with collateral vessel formation. Mild aneurysmal dilation of the ascending thoracic aorta was also observed. Additionally, multiple hilar and portacaval lymphadenopathies were identified, accompanied by hepatomegaly (21 cm) and splenomegaly (12 cm). Positron emission tomography (PET) scan (Biograph20_mCT, Siemens) showed multiple intensely hypermetabolic lymphadenopathies in both supra-diaphragmatic and infra-diaphragmatic regions. Hypermetabolic reticular opacities were present in both lung bases, along with a hypermetabolic nodule in the apex of the left lung, with findings suggestive of pulmonary involvement. Diffuse and intense hypermetabolic involvement of the bone marrow was present, particularly in the sternum, pelvis, and spine, with diffuse hypermetabolism in the spleen. These findings were primarily suggestive of a lymphoproliferative disorder (Fig. 1).

**Table 1:** Laboratory test results at presentation.

Test	Result	Reference range
Hb	9.8	12–15.5 g/dL
MCV	61	80–100 fL
Ferritin	19	20–200 ng/mL
ESR	78	0–20 mm/h
CRP	111	<10 mg/L
WBC	2700	4.000–10,000/mm^3^
SGOT	43	<31 U/L
SGPT	53	<33 U/L

CRP = C-reactive protein, ESR = erythrocyte sedimentation rate, Hb = hemoglobin, MCV = mean corpuscular volume, SGOT = serum glutamic oxaloacetic transaminase, SGPT = serum glutamic pyruvic transaminase, WBC = white blood cell count.

**Figure 1.**
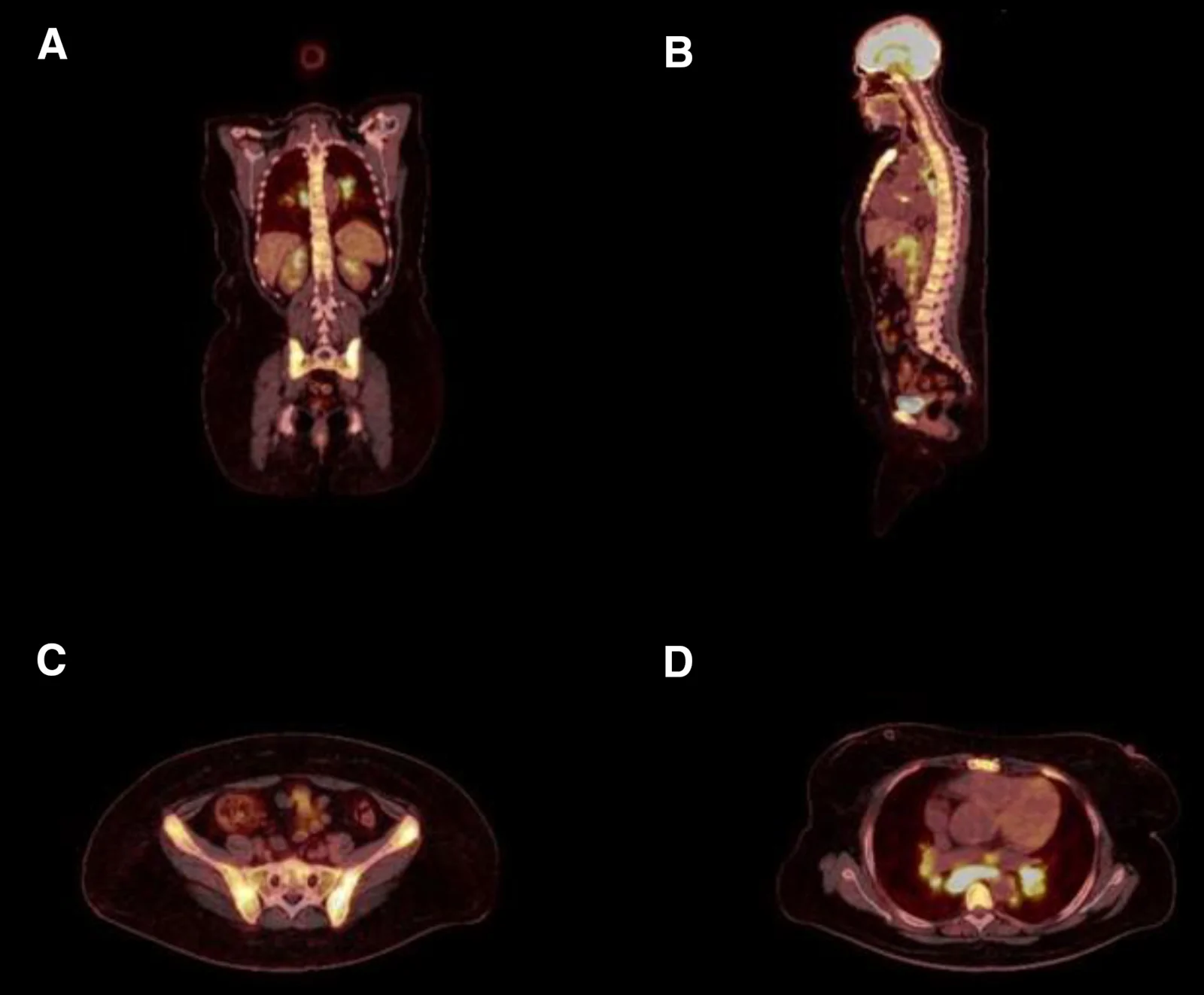
PET scan at presentation showing increased uptake in the perihilar region and the aorta (A, D), in the spine mainly the thoracic spine, sternum (B), and in the sacrum and iliac bones (C). PET = positron emission tomography.

Bone marrow biopsy showed 40% cellularity with maturing trilineage hematopoiesis and multiple interstitial and juxtatrabecular non-necrotizing granulomas. Megakaryocytes were present and morphologically unremarkable. The aspirate was hemodilute, with adequate touch preparations demonstrating maturing granulocytic and erythroid lineages. Blasts were scattered and accounted for 1% of nucleated cells. No stainable iron was identified. Acid-fast and Grocott’s methenamine silver (GMS) stains showed no definite microorganisms. The original histopathological slides and images could not be retrieved from the outside institution despite repeated attempts. Therefore, the pathological findings are described in detail based on the available histopathological reports.

A transesophageal biopsy of a mediastinal lymph node revealed non-necrotizing granulomatous inflammation, confirming the diagnosis of sarcoidosis. Concurrently, serum angiotensin-converting enzyme (ACE) level was elevated at 120 U/L (range < 65 U/L).

The patient was started on infliximab (Remicade, Janssen Biotech, Horsham) 300 mg given intravenously followed by subsequent doses at weeks 2 and 6, then every 8 weeks. Mycophenolate mofetil 2 g daily was maintained. Tocilizumab was discontinued because infliximab has a more established evidence base for the treatment of sarcoidosis, including data from randomized clinical trials and real-world observational studies. A follow-up PET scan was performed after the seventh dose of infliximab to assess the therapeutic response. It demonstrated partial improvement in the supra-diaphragmatic lymphadenopathy and near-complete regression of the infra-diaphragmatic lymphadenopathy. Hypermetabolic reticular opacities at the lung bases remained stable, while the hypermetabolic nodule in the apex of the left lung showed regression. Non-hypermetabolic micronodules scattered throughout both lung fields were stable. The intense and diffuse hypermetabolism of the bone marrow decreased markedly; however, splenic hypermetabolism increased. No hypermetabolic activity suggestive of active vasculitis was detected (Fig. 2). In addition to the improvements seen on the PET scan, the patient’s laboratory results improved significantly with infliximab treatment. Hemoglobin levels increased, while C-reactive protein (CRP) and erythrocyte sedimentation rate (ESR) decreased. Concurrently, the ACE level dropped markedly from the baseline value. The notable improvements in the patient’s laboratory parameters are summarized in Table 2. Written informed consent was obtained from the patient.

**Table 2:** Evolution of laboratory test results during treatment with infliximab.

Lab tests	Baseline results before initiating infliximab	Results after the seventh dose of infliximab	Range
Hb	9.8	11.2	12–15.5 g/dL
ACE	120	59	<65 U/L
ESR	78	23	0–20 mm/h
CRP	111	1.9	<10 mg/L
WBC	2700	5580	4000–10,000/mm^3^
SGOT	43	20	<31 U/L
SGPT	53	24	<33 U/L

ACE = angiotensin converting enzyme, CRP = C-reactive protein, ESR = erythrocyte sedimentation rate, Hb = hemoglobin, SGOT = serum glutamic oxaloacetic transaminase, SGPT = serum glutamic pyruvic transaminase, WBC = white blood cell count.

**Figure 2.**
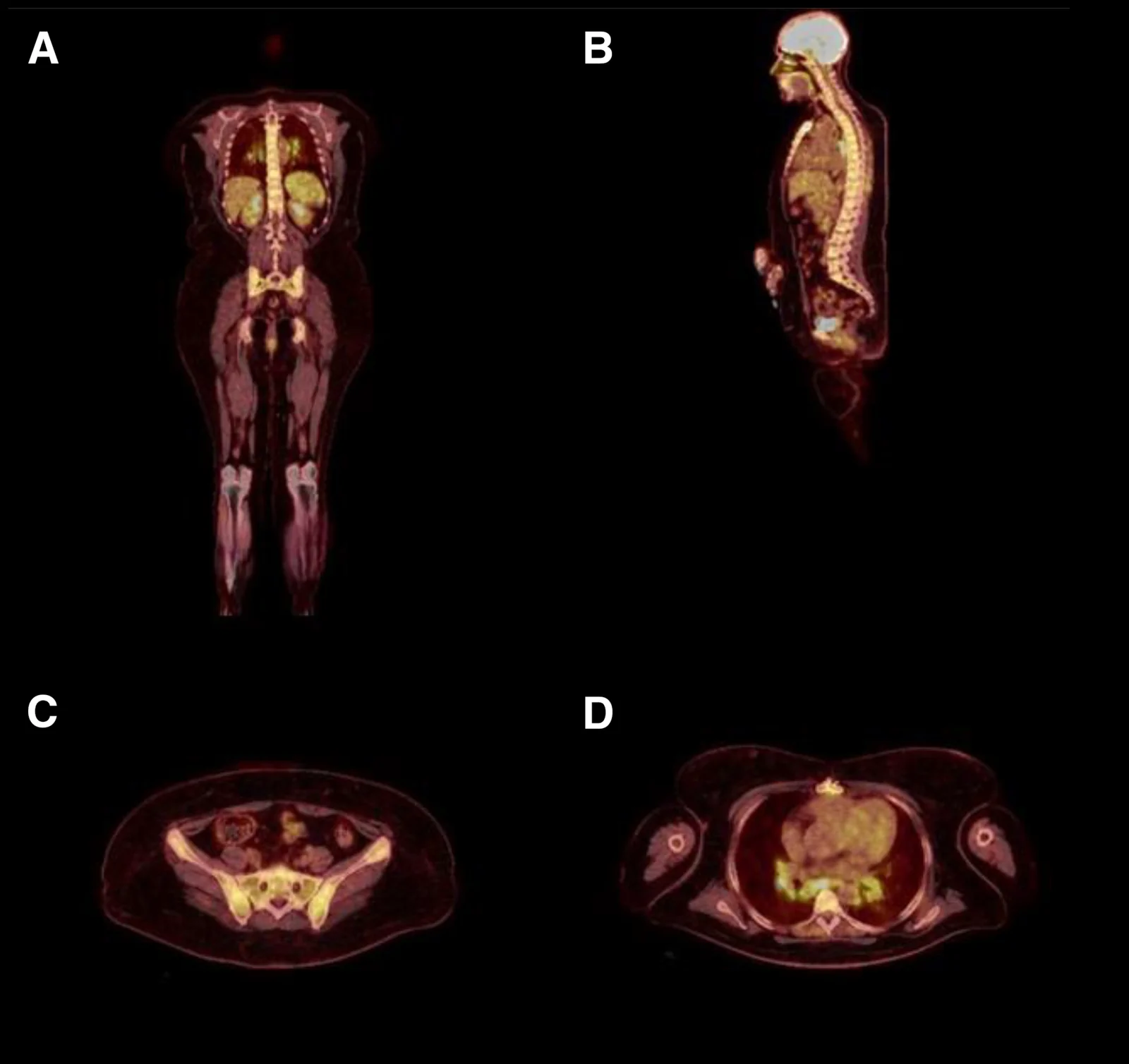
Second PET scan after seventh course of infliximab infusion showing reduced uptake in the thoracic spine, sternum and lungs (A,B,D) and in the sacrum and iliac bones (C). PET = positron emission tomography.

## 3. Discussion

TA and sarcoidosis share overlapping features such as granulomatous inflammation, elevated acute phase reactants, and multiorgan involvement, which can complicate diagnosis when these conditions coexist. Churg et al reported cases in which granulomatous inflammation affected both vascular and non-vascular tissues, suggesting a shared immunopathogenic mechanism.^[6]^ In our patient, key findings, such as hypermetabolic involvement of the lymph nodes, bone marrow, and spleen on imaging, alongside non-necrotizing granulomas on biopsy, were crucial in establishing the diagnosis of sarcoidosis and distinguishing systemic granulomatous inflammation from active TA-related vasculitic disease.

T helper 17 cell (Th17) lymphocytes are involved in TA. Singh et al identified elevated levels of T helper 17.1 cell (Th17.1) and programmed cell death protein 1 (PD1)-positive Th17 cells in patients with active TA.^[7]^ Th17.1 cells, characterized by the secretion of both interleukin-17A (IL-17A) and interferon-gamma (IFN-γ), are associated with corticosteroid resistance due to their expression of P-glycoprotein. Additionally, PD1-positive Th17 cells secrete transforming growth factor-beta 1 (TGF-β1), contributing to tissue fibrosis. These immune mechanisms, particularly Th17-related pathways and granulomatous inflammation, may represent shared immunological features of TA and sarcoidosis.

A 2019 review by Saha et al identified 25 reported cases of concomitant TA and sarcoidosis. Sarcoidosis preceded TA in 52% of cases, was diagnosed simultaneously in 24%, and followed TA in the remaining 24%.^[8]^ Similarly, in their 7-patient series, Chapelon-Abric et al reported that sarcoidosis preceded or coincided with TA in 86% of cases. Sarcoidosis generally had a classical, non-severe presentation and did not require specific treatment in 86% of patients.^[9]^ Our case is distinctive not simply because of the rarity of this association, but because of the exceptionally long interval of approximately 17 years between the diagnosis of TA and the subsequent development of sarcoidosis, together with the extent of organ involvement. Saha et al reported thoracic involvement in 60% and extrathoracic involvement in 32% of cases, with extrapulmonary manifestations affecting various organs.^[8]^ In our patient, sarcoidosis involved the lungs, spleen, and bone marrow, with non-necrotizing granulomas confirmed histologically in the bone marrow. Although osseous sarcoidosis has previously been described, bone marrow involvement was not documented in the cases summarized by Saha et al.^[8]^ The combination of delayed-onset sarcoidosis, multisystem involvement, and histologically confirmed bone marrow disease therefore represents the principal distinguishing feature of this case. Another noteworthy aspect is that sarcoidosis developed during ongoing treatment with tocilizumab and mycophenolate mofetil for TA. Although neither agent constitutes established standard therapy for sarcoidosis, this observation illustrates that new granulomatous disease may emerge in patients receiving immunosuppression for an associated inflammatory condition.

The associated anemia, leukopenia, hepatosplenomegaly, and increased bone marrow uptake on PET imaging further underscore the clinical significance of the systemic involvement. Although iron deficiency may have contributed to the anemia, the overall presentation warranted investigation for bone marrow involvement and exclusion of alternative diagnoses, particularly hematological malignancies. Granulomas are identified in approximately 0.3% to 2.2% of bone marrow biopsies, with sarcoidosis accounting for approximately 21% of biopsies showing granulomas.^[10]^ This case highlights the importance of reconsidering the differential diagnosis when new hematological abnormalities and multisystem manifestations arise in patients with longstanding TA, even after a prolonged interval and during immunosuppressive treatment.

Advanced imaging modalities such as PET can be useful in characterizing the distribution and pattern of inflammatory activity and, when interpreted in the appropriate clinical context, may help distinguish active vasculitic disease from systemic granulomatous inflammation. Betancourt et al highlighted the utility of PET imaging in a case report of sarcoidosis with concomitant Takayasu arteritis. Fluorodeoxyglucose (FDG) uptake patterns in both large vessels and lymphoid tissues enabled the identification of the dual pathology.^[11]^ Similarly, in our patient, widespread metabolic activity involving lymph nodes, bone marrow, lungs, and spleen was atypical for isolated TA and raised suspicion of a concomitant systemic granulomatous disorder.

Although increased aortic uptake was reported on baseline PET imaging, the extensive hypermetabolic activity involving the lymph nodes, lungs, spleen, and bone marrow, together with the histological demonstration of non-necrotizing granulomas and elevated serum ACE levels, supported the diagnosis of systemic sarcoidosis. Following infliximab treatment, repeat PET imaging showed no hypermetabolic activity suggestive of active vasculitis, accompanied by improvements in inflammatory markers and serum ACE levels. Taken together, these findings support systemic sarcoidosis as the predominant driver of inflammation at the time of treatment modification, although concomitant low-grade vascular inflammation at baseline could not be definitively excluded.

Our patient was receiving tocilizumab and mycophenolate mofetil, 2 immunosuppressive agents used in the treatment of TA.^[12]^ However, the emergence of systemic sarcoidosis during ongoing treatment prompted a reassessment of the therapeutic strategy. The efficacy of tocilizumab and mycophenolate mofetil in sarcoidosis remains insufficiently established, and treatment decisions should be individualized according to organ involvement, disease severity, and clinical course. In a study involving 64 patients with bone sarcoidosis, Zhou et al suggested considering infliximab in patients with aggressive or refractory disease.^[13]^ Sakkat et al likewise reported favorable outcomes with infliximab in a study of 33 patients with refractory sarcoidosis, particularly in patients with neurological and cutaneous manifestations.^[14]^ However, evidence specifically addressing the treatment of bone marrow sarcoidosis remains limited. Infliximab has also demonstrated efficacy as a steroid-sparing treatment for refractory TA,^[15]^ making it a potential therapeutic option when both conditions coexist. Accordingly, mycophenolate mofetil was maintained, while tocilizumab was discontinued and replaced with infliximab to target the predominant sarcoid inflammation. The subsequent clinical, laboratory, and imaging improvements support the potential utility of this treatment strategy. Nevertheless, the contribution of infliximab to the improvement of each disease process cannot be established definitively in this single case.

Finally, given the rarity of this condition and the inherent limitations of a single-case report, the generalizability of these findings, particularly regarding treatment response, remains limited. This case may nevertheless provide useful clinical insight when similar presentations are encountered.

## 4. Summary

The coexistence of TA and sarcoidosis is exceedingly rare and presents considerable diagnostic and therapeutic complexities. This case, to our knowledge the first to report bone marrow involvement by sarcoidosis in a patient with concomitant Takayasu arteritis, contributes to the expanding but still limited literature on the simultaneous occurrence of TA and sarcoidosis. It highlights the crucial role of combining advanced imaging techniques with histopathological analysis for an accurate diagnosis. Moreover, it underscores the importance of maintaining a broad differential diagnosis when patients with established inflammatory diseases develop new systemic manifestations that cannot be readily attributed to the underlying disease.

## Author contributions

**Conceptualization:** Oussama Fakih, Sara Hachem, Racha Chamas, Sally Abi Dargham, Ouidade Aitisha Tabesh, Fouad Fayad.

**Supervision:** Fouad Fayad.

**Validation:** Oussama Fakih, Ouidade Aitisha Tabesh, Fouad Fayad.

**Writing – original draft:** Oussama Fakih, Sara Hachem, Racha Chamas, Sally Abi Dargham, Ouidade Aitisha Tabesh, Fouad Fayad.

**Writing – review & editing:** Oussama Fakih, Sara Hachem, Racha Chamas, Sally Abi Dargham, Ouidade Aitisha Tabesh, Fouad Fayad.
